# Editorial Comment: Identification of Recurrent Anatomical Clusters Using Three-dimensional Virtual Models for Complex Renal Tumors with an Imperative Indication for Nephron-sparing Surgery: New Technological Tools for Driving Decision-making

**DOI:** 10.1590/S1677-5538.IBJU.2022.05.04

**Published:** 2022-08-03

**Authors:** Luciano A. Favorito, Natasha T. Logsdon

**Affiliations:** 1 Universidade do Estado do Rio de Janeiro Rio de Janeiro RJ Brasil Unidade de Pesquisa Urogenital - Universidade do Estado do Rio de Janeiro - Uerj, Rio de Janeiro, RJ, Brasil

Daniele Amparore ^1^, ^2^, Federico Piramide ^1^, Angela Pecoraro ^1^, ^2^, Paolo Verri ^1^, Enrico Checcucci ^3^, ^4^, Sabrina De Cillis ^1^, Alberto Piana ^1^, Giovanni Busacca ^1^, Matteo Manfredi ^1^, Cristian Fiori ^1^, Francesco Porpiglia ^1^

Eur Urol Open Sci. 2022 Mar 4;38:60-66

## COMMENT

In this interesting paper the authors shows the importance of the 3-D virtual models (3DVMs) to the study of kidney anatomy and the renal tumors before the nephron-sparing surgery (NSS). The robotic surgery provided important advances for the realization of the NSS, but the tumor anatomy is the key point of this procedure. Recently several papers shows the importance of the study of renal and ureteral anatomy with 3D printing models and CT-reconstruction models for surgical training for urological procedures (1–3). In the present paper the authors studied three patients with high-complexity renal masses with unusual anatomy and an imperative indication for NSS underwent contrast-enhanced computed tomography from which a 3DVM was obtained. The paper has amazing pictures of the kidney tumors anatomy. The authors concluded that three-dimensional models help in defining the best surgical strategy for kidney tumors, especially for complex tumors that require surgery to spare as much of the kidney as possible.

## References

[1] Marroig B, Frota R, Fortes MA, Sampaio FJ, Favorito LA. Influence of the renal lower pole anatomy and mid-renal-zone classification in successful approach to the calices during flexible ureteroscopy. Surg Radiol Anat. 2016;38:293-7. 10.1007/s00276-015-1562-0 26438274

[2] Sobrinho ULGP, Sampaio FJB, Favorito LA. Lower pole anatomy of horseshoe kidney and complete ureteral duplication: Anatomic and radiologic study applied to endourology. Int Braz J Urol. 2022;48:561-8. 10.1590/S1677-5538.IBJU.2022.99.12 PMC9060160 35333487

[3] Sobrinho ULGP, Albero JRP, Becalli MLP, Sampaio FJB, Favorito LA. Three-dimensional printing models of horseshoe kidney and duplicated pelvicalyceal collecting system for flexible ureteroscopy training: a pilot study. Int Braz J Urol. 2021;47:887-9. 10.1590/S1677-5538.IBJU.2021.99.10 PMC8321488 33848082

